# IMERGE-FEP: Improving Relative Free Energy Calculation Convergence with Chemical Intermediates

**DOI:** 10.1021/acs.jpcb.4c07156

**Published:** 2025-02-20

**Authors:** Linde Schoenmaker, Daan A. Jiskoot, Jenke Scheen, Evien Cheng, Vytautas Gapsys, David F. Hahn, Benjamin Ries, Gerard J.P. van Westen, David L. Mobley, Willem Jespers

**Affiliations:** † Leiden Academic Centre for Drug Research, Leiden University, Einsteinweg 55, 2333 CC Leiden, The Netherlands; ‡ Department of Pharmaceutical Sciences, University of California, Irvine, California 92697, United States; § Open Molecular Software Foundation, Davis, California 95618, United States; ∥ Computational Chemistry, Janssen Research and Development, Janssen Pharmaceutica N. V., Turnhoutseweg 30, 2340 Beerse, Belgium; ⊥ Medicinal Chemistry, Boehringer Ingelheim Pharma GmbH & Co KG, Birkendorfer Str 65, 88397 Biberachan der Riss, Germany; # Open Free Energy, Open Molecular Software Foundation, Davis, California 95616, United States

## Abstract

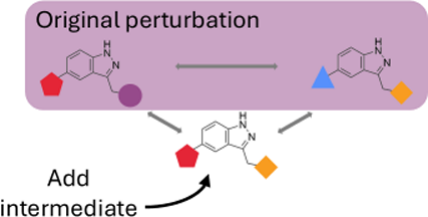

Alchemical free energy calculations are becoming an increasingly prevalent tool in drug discovery efforts. Over the past decade, significant progress has been made in automating various aspects of this technique. However, one aspect hampering wider application is the construction of perturbation networks to connect ligands of interest. More specifically, ligand pairs with large dissimilarities should be avoided since they can lower convergence and decrease accuracy. Here, we propose a technique for automatic generation of intermediate molecules to break up problematic edges—calculations connecting two different ligands or molecules—into smaller perturbations. To this end, a modular tool was developed that generates intermediates for a molecule pair by enumerating R-group combinations called IMERGE-FEP (Intermediate MolEculaR GEnerator for Free Energy Perturbation). Intermediate enumeration of multiple, representative congeneric series showed that intermediates increase similarity regarding shared substructures, geometry, and LOMAP scores. Taken together, this tool eases integration of intermediate steps into free energy calculation protocols.

## Introduction

A large part of early stage drug discovery consists of finding and optimizing hit compounds. This involves the enhancement of ligand-target binding and pharmacokinetic properties. To make this process more efficient, computational techniques can be employed. One such technique is Free Energy Perturbation (FEP), which can provide high accuracies, generally in the range of 1–2 kcal/mol, at reasonable computational cost, provided that care is taken in the setup of the protein–ligand system.^1−3^ FEP can be used to calculate differences in free energies by employing thermodynamic cycles. Examples include the calculation of hydration free energies, by representing the molecule in gas state and solvated state, or binding affinity by comparing the solvated (unbound) and protein bound states.

This work focuses on relative free energy calculations and how to improve the applicability of this technique.^4^ For a reliable free energy estimate, the phase space of the two end states need to sufficiently overlap.^5,6^ If this is not the case, the free energy will be slow to converge, or worse, potentially lead to less accurate predictions. Commonly to increase phase-space overlap, transformations are performed using multiple nonphysical intermediate states. These states connect the two end states and consist of a combination of the interacting elements from both ligands according to the lambda variable, e.g., in an intermediate state 90% of the interactions of ligand A and 10% of those of ligand B can be present.^7^ Since this approximation made for modeling cannot physically exist, intermediate states are often also referred to as “alchemical” intermediates.

In most free energy calculation applications, a set of ligands is investigated to establish a ligand ranking, and in order to obtain binding free energies of all ligands of interest, a network of calculated free energy differences between different ligands needs to be defined that spans the whole graph of ligands being considered. Because ligand similarity affects prediction accuracy the choice of which ligands to compare is important. Therefore, one of the challenges when doing a free energy calculation for a set of ligands is constructing the optimal network. The molecules being perturbed need to share enough phase space overlap, such that one can accurately calculate the changes in free energy. In addition to this, an optimal network should include as few redundant transformations as possible, while also allowing some amount of redundancy for error detection/recovery. In this regard, extensive benchmark sets, atom mapping tools-like LOMAP-, and recently data-driven network generators have been pushing the field forward.^2,8−10^

Nonetheless, arriving at a final network for a ligand series typically still involves manual curation and editing.^11^ This, however, is inconvenient and quickly becomes infeasible as the number of ligands of interest increases. The main issue that can arise is the inclusion of difficult to converge edges in the network. When these are identified an alternative path can be constructed. Or alternatively, adding an intermediate molecule between the molecules of the inaccurate edge can improve performance. By introducing an intermediate that resembles both original end points, a large perturbation can be broken up into multiple perturbations. Previous work by Boresch and Bruckner has shown the successful application of atom-wise perturbations for computing free energy differences.^12^ Another example is the 2020 publication by Kuhn et al. in which several intermediate structures are added manually to perturbations involving changes in ring size and scaffold hopping.^13^ In addition to this, Flare FEP by Cresset, offers the functionality to add intermediate molecules to a network. Another more recent tool, called PairMap, iteratively generates a pool of intermediates on a per-atom basis, and uses those to create an optimal network.^14^

Here we set out to create a modular, open-source method for obtaining chemically meaningful intermediates that are a combination of the two original end points, with the specific goal to generate a set of molecules between each parent pair that are more similar to both parent molecules than the parent molecules are to each other. This way, suitable intermediates can be selected and applied to create perturbations with more phase space overlap, possibly improving convergence.

This study presents a tool for the automatic generation of chemical intermediates, called IMERGE-FEP (Intermediate MolEculaR GEnerator for Free Energy Perturbation). It generates intermediates in a pairwise manner, using the maximum common substructure (MCS) and enumerated substituent combinations. Each intermediate’s suitability is evaluated based on its similarity to its two parent molecules. In addition to this, the application of intermediates for FEP is shown on a series of ligands using relative hydration free energy (RHFE) and relative binding free energy (RBFE) calculations with hybrid topology, focusing on energetic convergence over time.

## Methods

### Intermediate Generation

Several design goals were established for the automatic generation of intermediates. Most importantly, a good intermediate should be more similar to both parent molecules than the parent molecules are to one another. The way we chose to achieve this is by having the core of the intermediate be the MCS of the parent molecules. Intermediates are then formed by attaching the parent molecules’ side-chains to the common core.

Next, intermediates are generated using a relatively simple algorithm that quickly yields potentially interesting molecules. Afterward, these molecules should be narrowed down to the most suitable candidates. To this end, we included a pruning algorithm that can rank intermediates based on the following properties; Tanimoto similarity, LOMAP score, ROCS score (OpenEye Scientific Software, combination of shape and color) and the number of heavy atoms.^8,15,16^ The latter compares substituents and allows the user to filter out intermediates with R-groups with a number of heavy atoms above or below a certain threshold. For the other scores, the intermediate is by default compared to both parents and the resulting scores can be combined using the sum, weighted sum, and harmonic mean (optionally after min–max normalization).

In addition to this, to meet (potentially) different intermediate generation requirements, the code was made modular to facilitate changing aspects of the generator. This allows the user to easily adapt the pruning to their own needs. It also makes it possible to change the MCS search algorithm or to, for example, expose the table of R-groups to be attached to the core and apply certain filters or extend these R-groups by means of *de novo* generation methods.

**Figure 1:**
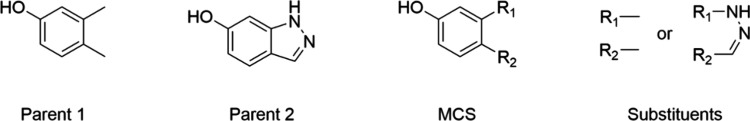
Example of a molecule pair with a fused ring system. Parents: input molecules for the intermediate generator. MCS: maximum common substructure. Substituents: R-groups that will be attached to the MCS.

Finally, multiple decisions were made on the level of the molecular structure. Since stereoisomerism influences the outcome of the MD simulation, it is important to conserve the chiral information on the molecules of interest. Molzip, the existing method for recombination provided by RDKit, does not preserve this information.^17^ Hence a similar functionality to molzip was made with different RDKit modules, as explained in more detail below. In addition to this, the decision was made to not alter rings fused to the MCS, but instead treat the involved substituents as one substituent as shown in Figure 1. Hence,the fused ring is left intact by not changing substituent position R1 and R2 independently from each other. This way, no new ring breaks are introduced. This was done because ring breaking can be problematic for single and hybrid topology approaches, and often requires more time to converge or can even introduce thermodynamic cycles which fail to close.^18^

Apart from creating new molecules via recombination of R-groups, the original parent molecules are also reconstructed by connecting their substituent groups back to the core. When this recombination does not lead to the original molecules, the pair is flagged to highlight a problem in the intermediate generation for that specific pair.

**Figure 2:**
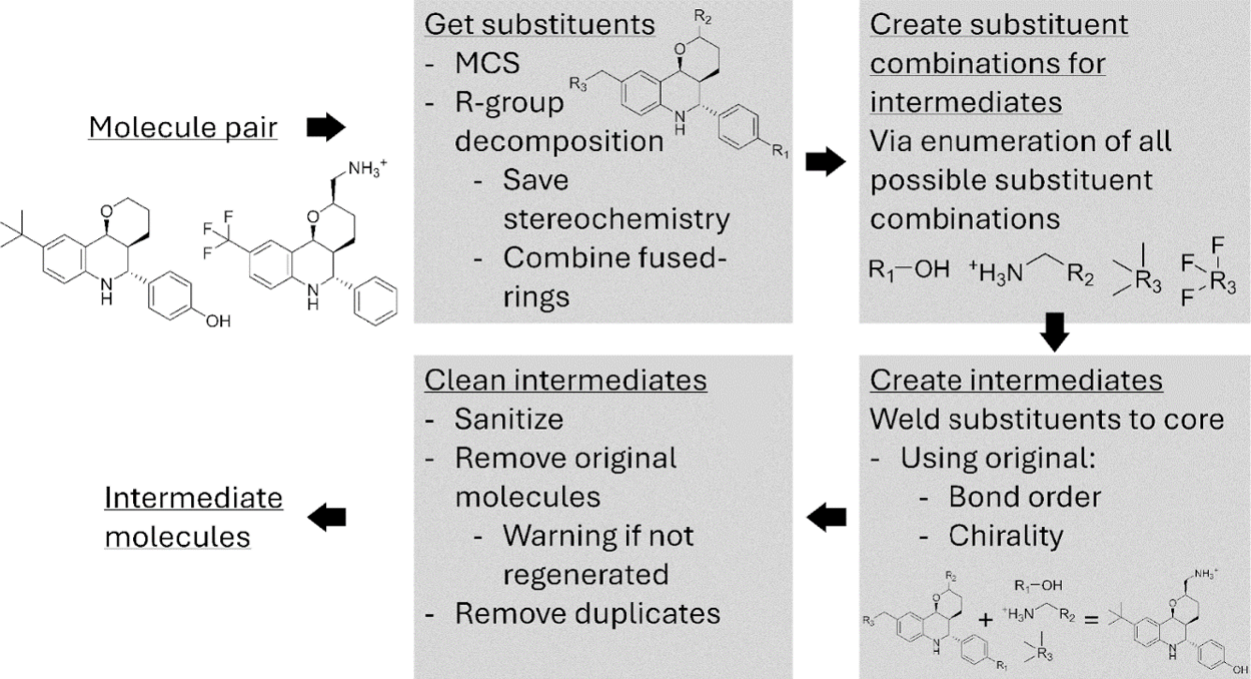
Overview of the intermediate generator workflow.

The criteria described above are combined to create the R-group enumerator, see Figure 2. Briefly, the MCS of the parent molecules is found and based on this core a table with R-groups is constructed. The R-groups are enumerated and attached back onto the core. After sanitization, additional pruning steps can be applied. The intermediate generation algorithm has been implemented in Python 3.12. The libraries used include RDKit 2023.09.1 and optionally OpenEye for ROCS scoring. It is available at https://github.com/CDDLeiden/RGroupInterm.^16,17^

### Data Set

**Figure 3:**
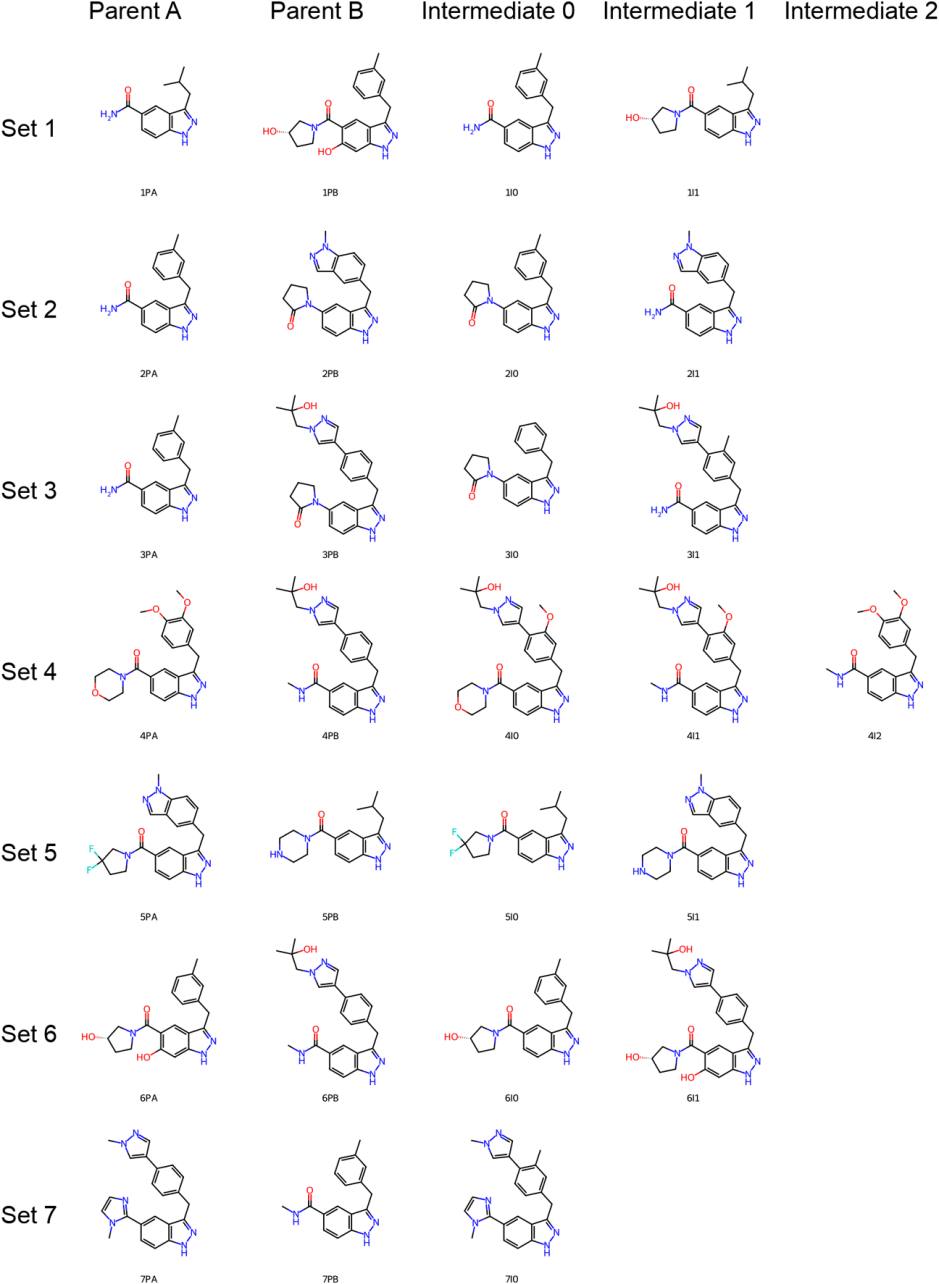
Molecular structures of parent pairs and generated intermediates tested in FEP simulations. Parent: molecule used as input for the intermediate generator. Intermediate: generated intermediate.

The aim of the intermediate generation presented here is to break up an individual free energy perturbation into two smaller perturbations. To study the effect of intermediate generation on similarity, congeneric series from the Protein–Ligand Benchmark Data set and the Benchmark set for relative free energy calculations were used.^2,19^ It is beyond the scope of this work to test whether such a separation will improve overall efficiency, but that is a hypothesis we hope to further test in a subsequent work (specifically, that the total computational effort required to converge two “straightforward” perturbations to a specified precision may be less than the total computational effort required to similarly converge a single “difficult” perturbation). For a preliminary investigation of the effect of intermediates in FEP simulations, multiple sets of perturbations based on substituent combinations from the cyclin-dependent kinase 8 (CDK8) benchmark set from the Protein–Ligand Benchmark Data set were constructed (see https://github.com/CDDLeiden/IMERGE-FEP/blob/main/supplemental_information/perturbations.ipynb). This set was chosen because these molecules have a relatively small core, yet large variations on the R groups, which cover various alchemical transitions. In total 7 parent pairs were constructed for which intermediate structures were generated (Figure 3). The intermediates that were tested were selected based on having a high similarity to both parent molecules. The following naming convention was used; parent molecules are denoted by the set number, followed by the letter P for parent, followed by either A or B; intermediate molecules are denoted by the set number, followed by the letter I for intermediate, followed by the a unique number starting from 0.

For the FEP simulations, ligand coordinates were obtained via two methods. For the simulations in the protein bound state, the coordinates of the atoms in the MCS of the most similar molecule in the CDK8 benchmark set were used. For the other simulations, ligand coordinates were obtained by using the Merck molecular force field (MMFF) to optimize the conformation of a reference molecule for each set.^20^ The coordinates of the MCS were then restrained and conformers of the other molecules were generated and aligned.

### Simulation Methods

To show the application of chemical intermediates to FEP simulations, alchemical perturbations between pairs of ligands were performed. Ligands were parametrized with the Sage 2.1.1 force field.^21,22^ For the protein parametrization AMBER99sb*ILDN was used.^23−25^ Waters were modeled using transferable intermolecular potential with 3 points (TIP3P).^26^ A dodecahedral simulation box was used. For the water leg, the distance between solute and box wall was set to 2 nm. For the protein leg, the distance between protein and box wall was set to 2 nm and the charge of the protein was neutralized by adding ions. Calculations were carried out with GROMACS (version 2022.1) using PMX for creating hybrid topologies.^27,28^ The hybrid topology of a perturbation was created using the function LigandHybridTopology. The path between the end states consisted of turning off Coulombic interactions of initial state A, transforming Lennard-Jones interactions and then turning on Coulombic interactions of the final state B. To achieve this the hybrid topology was split into two hybrid topologies: one of state A with charges and state B without charges and one topology of state B with and without charges. Dummy atoms were decoupled as described by Fleck et al.^29^ Simulations were run using a stochastic dynamics integrator, where hydrogen bonds were constrained and the time step was set to 2 fs. Long-range Coulombic interactions in the water and protein leg were truncated using Particle Mesh Ewald (PME) at 1.2 nm.^30,31^ The temperature was set at 298.15 K and pressure was controlled using the Parrinello–Rahman barostat.^32^ Each transformation was run in triplicate. For each transformation the production simulation was run for 7.5 ns. In vacuum constant-temperature, constant-volume ensemble (NVT) equilibration was done for 1 ns. For the water and protein legs, NVT equilibration was done for 10 ps, followed by 1 ns of constant-temperature, constant-pressure ensemble (NPT) equilibration. For each transformation, charges of all atoms of initial state A were set to zero in 5 equidistant lambda states, this was followed by a transform of the Lennard-Jones interactions in steps of 0.1 and finished by adding the charges of end state B with a lambda step size of 0.25.

### Analysis

The free energy calculations were analyzed using Alchemlyb.^33−35^ In this paper the mean and standard deviation of 3 independent runs are reported. The standard deviation of the path via the intermediates is calculated as the root of the summed square of the standard deviations of both paths. To assess convergence, the Δ*G* was calculated every 0.1 ns and the threshold for convergence was set at a maximum change of 0.1 kcal/mol over 2 ns.

## Results

### R-Group Enumerator

**Figure 4:**
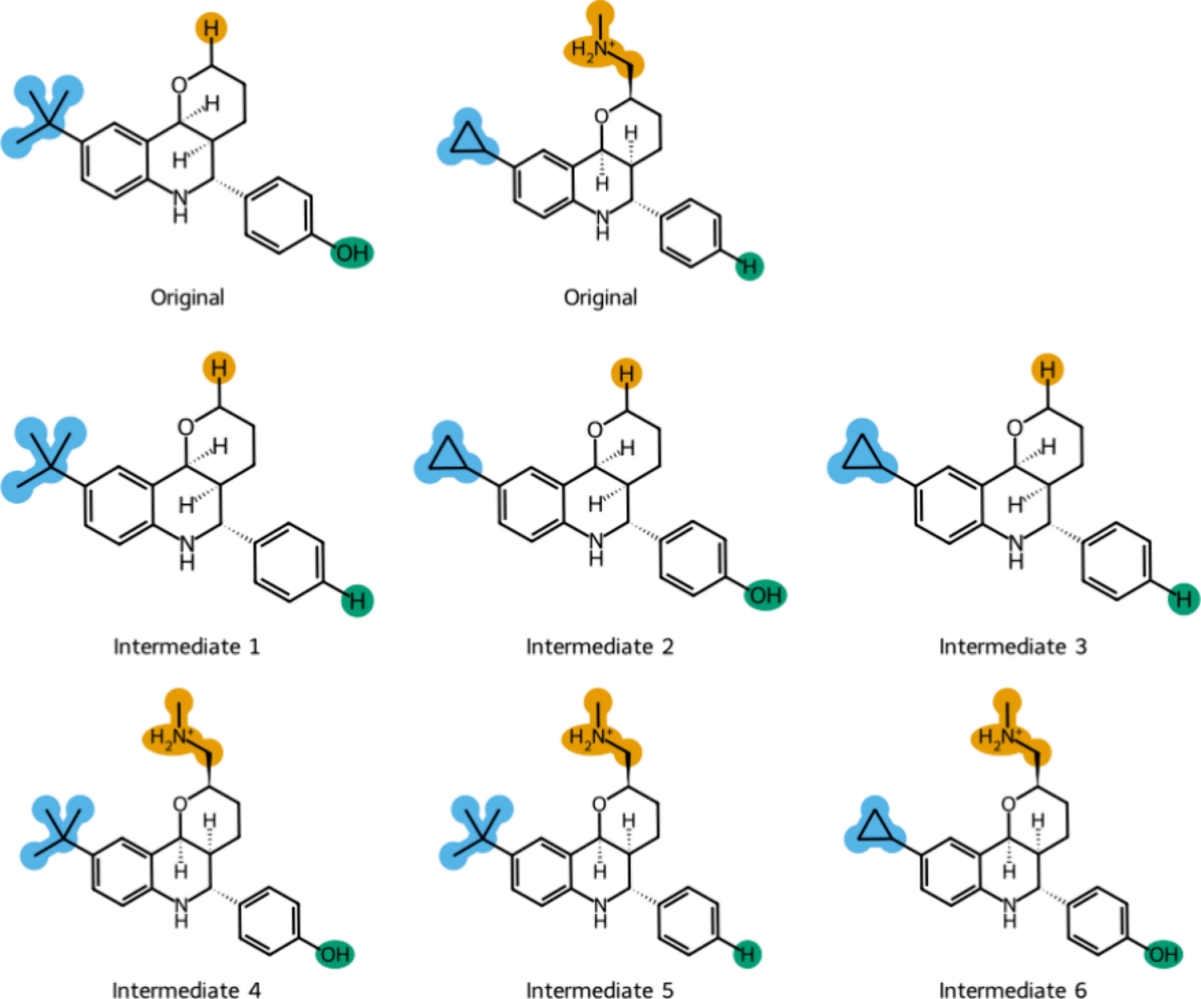
Example of intermediates generated for a pair of molecules from the Eg5 congeneric series. Original: input molecule for the intermediate generator. Intermediate: generated intermediate. Colors denote different substituent sites.

To establish that the generator yields useful intermediates, i.e., molecules that meet the established design goals, it was applied to the congeneric series in the Protein–Ligand Benchmark Data set and the Benchmark set for relative free energy calculations. Figure 4 shows an example from a pair of molecules from one of the protein targets in the benchmark set, Eg5. This pair has 3 R-group sites that differ, meaning that recombination yields 6 new molecules. This example shows that the chiral information on the shared core and attached functional groups is kept. Specifically at the site highlighted in orange, the secondary amine maintains its original orientation.

**Figure 5:**
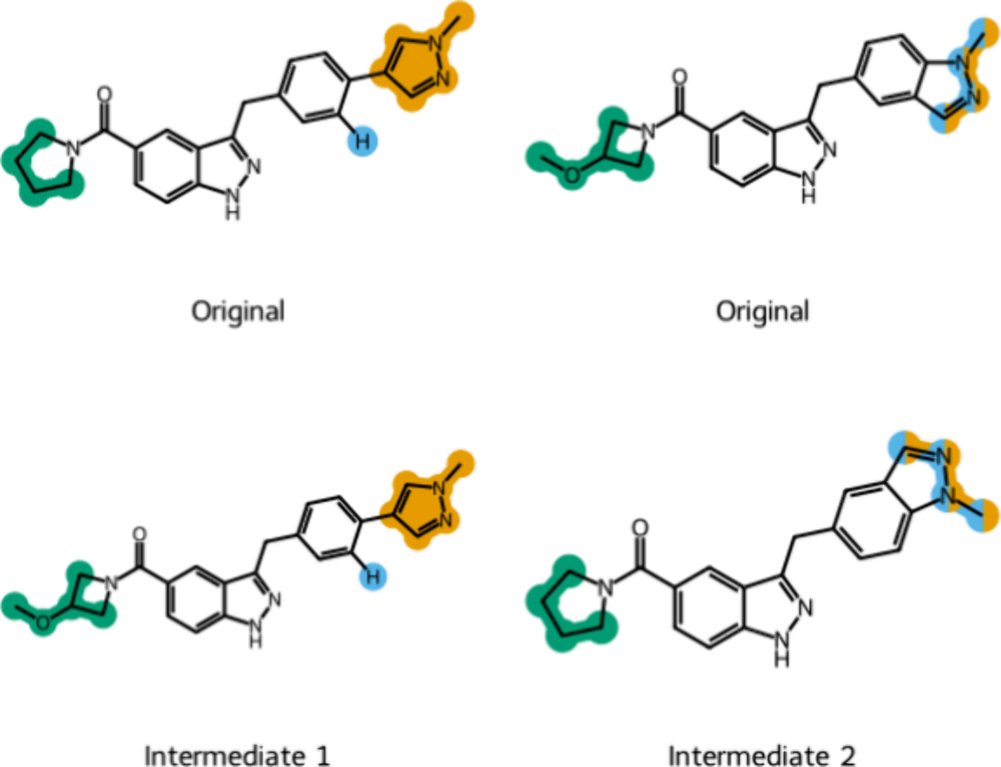
Example of intermediates generated for a pair of molecules with fused rings from the CDK8 congeneric series. Original: input molecule for the intermediate generator. Intermediate: generated intermediate. Colors denote different substituent sites.

A second characteristic of the intermediate generation that is specific to this implementation is that fused rings are not altered. In cases where the common substructure is part of a larger ring system for one or both parent molecules, the attached rings are not broken. Instead an attached ring is seen as 1 R-group as is shown in Figure 5 for the group highlighted in blue and orange.

Within the congeneric sets a total of 5369 unique parent combinations with two or more different R-groups can be made. For this work we applied the intermediate generation algorithm to all these pairs to test its outputs for different scaffolds and R-groups. R-group enumeration was successful for 97% of input pairs, as evaluated by the regeneration of the original inputs. In total this yielded 27,746 intermediates. The median run time for intermediate generation per pair is 0.1 s and the average is 0.6 s.

As an additional test, 5 molecules with redundant R-groups were left out of the Eg5 set, a congeneric series consisting of 28 ligands. After recombination of the remaining molecules these 5 molecules were found back in the intermediate set showing that plausible intermediates were generated. Together these results show that our algorithm can be applied to generate molecules that meet the design goals, and qualitative evaluation shows that these intermediates contain aspects of both parent molecules and therefore increase similarity.

### R-Group Enumeration Increases Similarity

**Figure 6:**
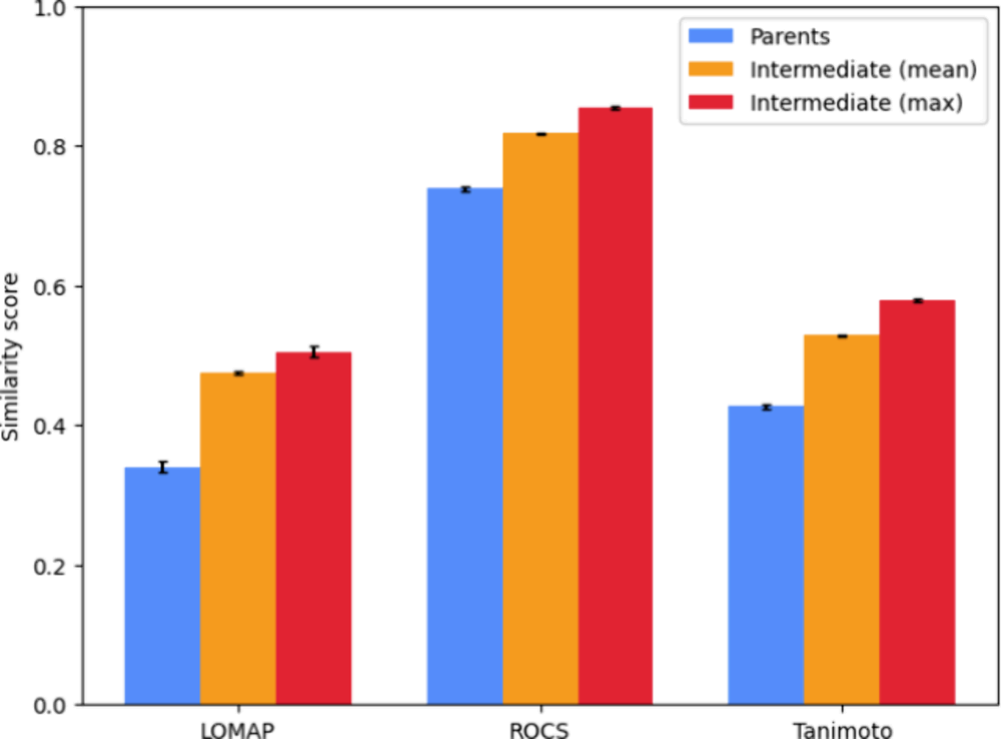
Similarity of intermediates compared to parents. Similarity was assessed using the LOMAP, ROCS and Tanimoto score. Parents: average similarity between original pairs. For intermediates the harmonic mean of the similarity score compared to both parents was calculated. Mean: the average of all intermediate scores. Max: the average of the most similar intermediates (based on the harmonic mean). Error bars show the 95% confidence intervals.

The generated intermediates were quantitatively assessed using multiple similarity metrics relevant for free energy calculation applications. As a baseline the average similarity of the parent pairs was calculated. For the intermediates the harmonic mean of the similarity to both parents is taken. As intermediate generation yields multiple intermediates per pair, the average and maximum value per parent pair was calculated before taking the mean over all pairs. Figure 6 shows the resulting similarity values. On average, the intermediates that are generated are more similar to both parents than the parent molecules are to each other. When solely looking at the best scoring intermediate per pair the similarity increases further. An especially large difference can be seen in the LOMAP score which increases from 0.34 to 0.51. These similarity scores show that the intermediate generator creates molecules that increase similarity to both parents.

### RHFE and RBFE Cycle Closure and Variance

**Figure 7:**
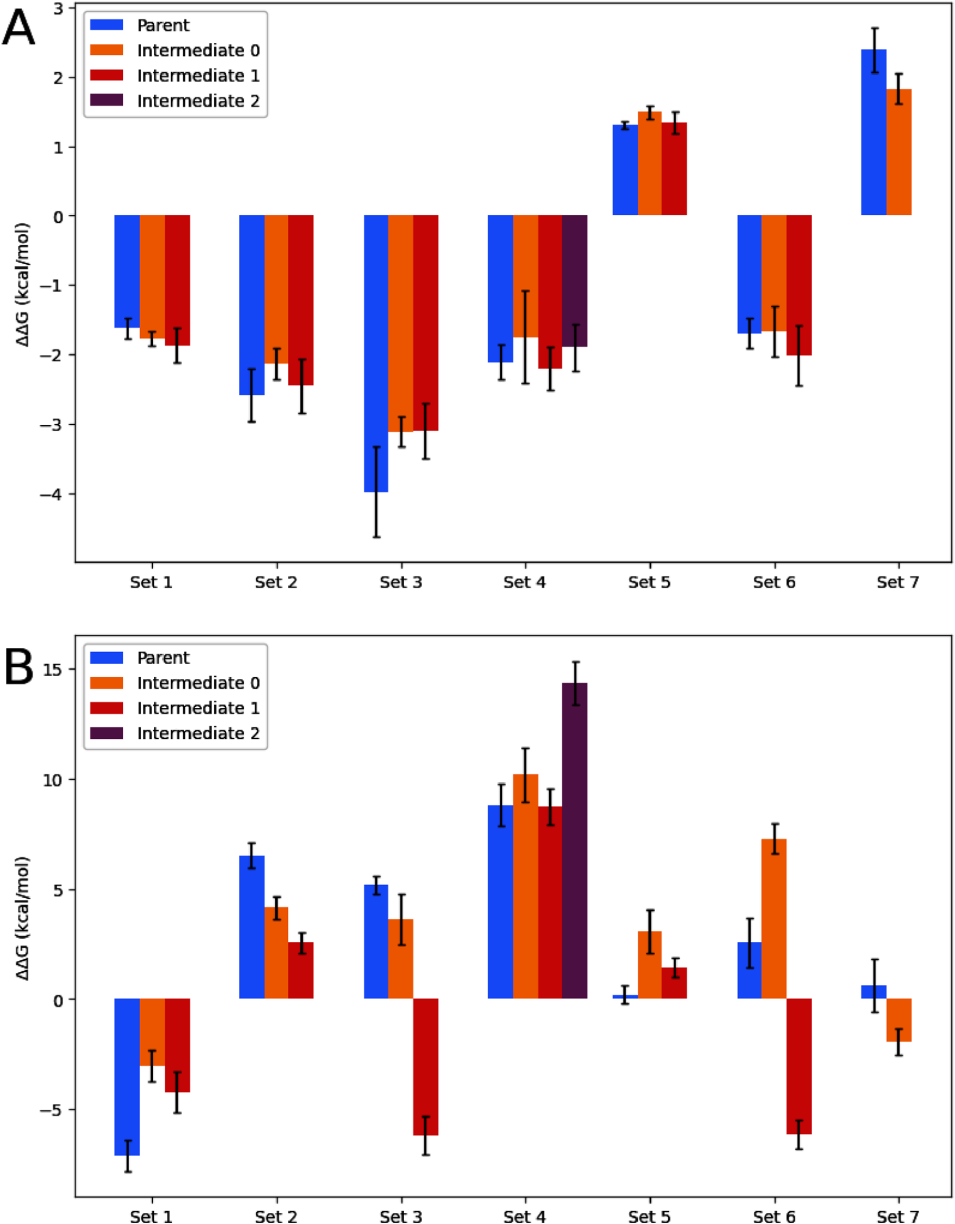
ΔΔ*G* of RHFE (A) and RBFE (B) transformations. Parent: ΔΔ*G* of transformation without intermediate step. Intermediate: summed ΔΔ*G* for transformation with added intermediate step, one intermediate per bar. Standard deviation is calculated over 3 replicates. The production run time of the parent run (7.5 ns) was equal to the combined run time of the two runs via the intermediate.

To compare the difference between the outcome of runs with and without intermediate steps, RHFE and RBFE transformations were run for 7 different sets of molecules. All sets consist of a direct transformation and one or multiple runs with the same end points and an intermediate step. The summary statistics of those transforms are shown in Figure 7. Free energies ought to be path-independent, and thus cycle closure is expected along the A → I → B path and A → B path, meaning they should add up to zero. For RHFE, in most cases the ΔΔ*G* values that are obtained for the end points do not significantly differ for the path with and without an intermediate. For intermediate 0 from set 5 and intermediate 0 from set 7, the ΔΔ*G* is significantly different from the ΔΔ*G* from the parent path. The exact differences for the first are small, at 0.17 kcal/mol, but for the latter larger at 0.7 kcal/mol. An explanation for the behavior seen in set 7 could be that that two rings are changed at the same time. The standard deviation of the transformations with an intermediate step is generally similar to or lower than the standard deviation of the direct transform. For set 1, 2, 3, and 7 at least one of the intermediate transforms has a lower standard deviation. For all other transforms, except for intermediate 4I0, the standard deviation is similar to the direct transform.

In case of the protein legs, starker differences between the parent and the intermediate paths are observed (Figure 7B) with fewer cycle closures. In some cases, different intermediate paths agree between one another, but not with their parent leg (Set 1, 2, 4, and 5). In other cases, the differences between intermediate paths are large (set 3 and 6). A main contributor to this behavior is the fact that the intermediate adopts different orientations in the binding site when going from A → I compared to B → I, and thus no cycle closure is to be expected. If this behavior is observed, we recommend to discard the simulation, or alternatively run perturbation B → I, as I → B by starting the simulation with the coordinates of the intermediate obtained from the A → I perturbation. As should be expected, standard deviations in the protein legs are larger than the protein and water legs, and in some cases do not meet convergence criteria (have a larger than 1 kcal/mol standard deviation), in which case we recommend to discard the calculation. However, the trend that intermediate paths generally have equivalent or lower standard deviations is maintained (see also SI Table 1). Finally, it is noteworthy to mention that many of these perturbations can be considered challenging even with an intermediate used.

These results show that – in RHFE – for most sets cycle closure holds and that for both RHFE and RBFE calculations, adding an intermediate step either improves or maintains the standard deviation, suggesting that adding the intermediate is an efficient way to use computational time. We cannot exclude however that in some cases simply running the simulations for longer instead of adding an intermediate might be a more efficient way of using computational time. Another option would be to use the sampling time to add additional replicate simulations of the system, starting the simulation using the coordinates of system B, rather than system A. In any case, adding intermediates is only efficient when these improvements are proportional to the added computational effort.

### Relation between Convergence and Molecular Similarity

**Figure 8:**
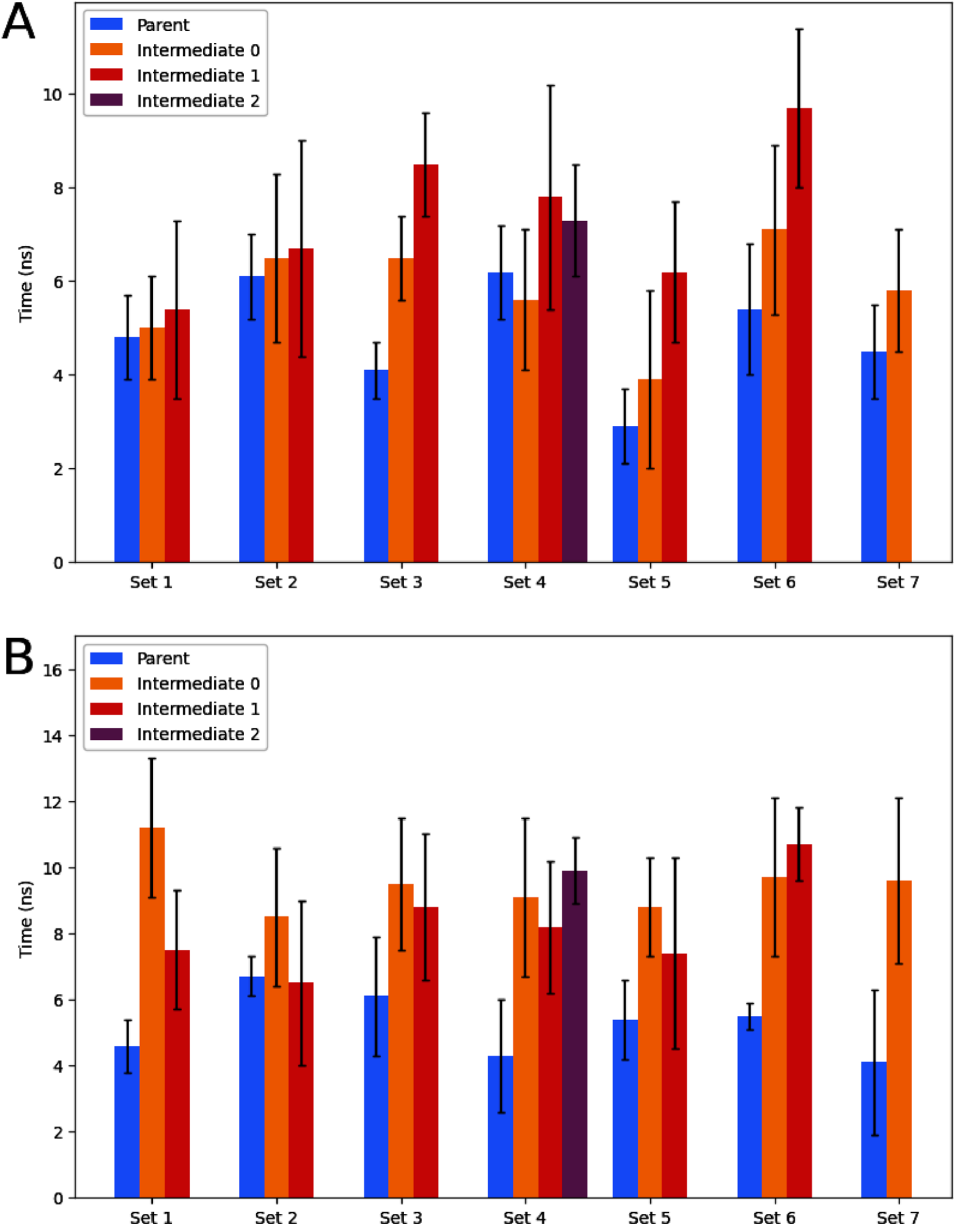
Time until convergence of the path between the two end points for the water leg (A) and protein leg (B) shows that the intermediate path takes less than twice as long to converge. Here convergence was said to be reached when the deviation in Δ*G* value was less than 0.1 over a period of 2 ns. Parent: time until convergence of transformation without intermediate steps. Intermediate: summed time until convergence for transformations with added intermediate step. Standard deviation is calculated over 3 replicates.

The change in Δ*G* over the simulation’s runtime was compared in order to examine the impact of an intermediate step on the time until convergence (Figure 8). In this work, convergence is defined as the time point after which the ΔΔ*G* changes a maximum of 0.1 kcal/mol over a period of 2 ns. Overall, the variance between replicates is relatively high. On average, for both the vacuum and water leg, the intermediate path takes roughly 20% longer to converge than the direct path. Interestingly, for the solvated system specific intermediates from set 1, 2, 4, and 7 take the same amount of simulation time to converge as the parent transformation. This indicates that the individual runs do converge faster, meaning that connecting the parent end states with an intermediate yields an additional data point at the same computational cost. This trend is partly maintained in the protein leg simulation times (set 2, 3, 5), however in other cases intermediate paths do not decrease the simulation time to reach convergence (1, 4, 6, and 7). Moreover, additional care needs to be taken to ensure intermediates sample the same conformational space. When this is not the case energetic differences between the direct parent and intermediate pairs can differ significantly (e.g., for set 3 and set 6).

## Conclusions

This study presents a novel method for automatic intermediate generation and sets out to evaluate the effects of using chemical intermediates on calculation performance. These results support the relevance of using chemical intermediates in regards to the improved convergence and the potential for higher accuracy.

Qualitative and quantitative analysis of the intermediate generator shows that it fulfills the design goals we set. The generation algorithm is based on a modular script and is able to generate intermediates keeping the stereoisomer orientation intact while not introducing ring breaks. Depending on the number of substituent positions a high number of intermediates can be generated. These intermediates have a higher similarity to both parents than the parents have to one another, as shown by multiple similarity metrics. Overall, we think that this tool will be useful for quick idea generation and a next step in automating the creation of optimal congeneric networks. Further enhancements could be implemented such as the introduction of different methods to identify the shared core of molecules, e.g., via 3D pose alignment.^36^ Additionally, intermediate substituents could be created using *de novo* generation tools. Steps could also be taken to address the protonation of the intermediate molecules and exclude charge changes *a priori*. Apart from further development on the generation side, pruning could be extended to, for example, find intermediate paths for very large perturbations.

Testing of a small set of routes via an intermediate compared to a direct control in RHFE shows that the final ΔΔ*G* values are comparable. This indicates that both paths likely give valid results. Comparing the propagated uncertainty of the intermediate path with the original large perturbation indicated that going via the intermediate path can decrease the uncertainty—though it should be noted that only a small number of perturbations were tested. More research is needed to establish in which cases intermediates will be beneficial to use. Comparing the time until convergence shows that the intermediate runs generally converge faster. On average, the intermediate path takes only 20% longer to converge than the direct path. This means that if an intermediate step is used for runs that stop at convergence, an additional data point can be obtained with relatively little additional computational resources. While this method might reduce the amount of simulation time needed, this work also shows that reaching sufficient phase-space overlap between dissimilar molecules remains challenging, and particular care needs to be taken to assess whether the intermediate paths share conformational space particularly in the protein legs. Taken together, these results hint at beneficial effects of including chemical intermediate steps into free energy networks and warrant more research into the topic. In addition, the process of adding intermediates can also aid in the design process itself, i.e., in some cases add novel ideas automatically in the ligand design campaign.

## Data Availability

All code is available on Gitub: https://github.com/CDDLeiden/IMERGE-FEP, input data is available on zenodo: https://zenodo.org/records/14639911.

## Supporting Information Available

Mean and standard deviation values of ΔG of 3 replicates run in protein, water, and vacuum; and mean and standard deviation values of RHFE and RBFE ΔΔG of 3 replicates (PDF)

## Abbreviations

CDK8: cyclin-dependent kinase 8
FEP: free energy perturbation
IMERGE-FEP: Intermediate MolEculaR GEnerator for Free Energy Perturbation
MCS: maximum common substructure
MD: molecular dynamics
MMFF: Merck molecular force field
NPT: constant-temperature, constant-pressure ensemble
NVT: constant-temperature, constant-volume ensemble
PME: Particle Mesh Ewald
RHFE: relative hydration free energy
TIP3P: transferable intermolecular potential with 3 points
