# Supplementary material for: IMERGE-FEP: Improving Relative Free Energy Calculation Convergence with Chemical Intermediates
Source: J Phys Chem B. 2025 Feb 20;129(9):2370–9. doi: 10.1021/acs.jpcb.4c07156 (PMC11891892; doi:10.1021/acs.jpcb.4c07156)
Supplement: Supplementary file 1 — jp4c07156_si_001.pdf [file jp4c07156_si_001.pdf]

Supplemental information - IMERGE-FEP: Improving relative free energy calculation convergence with chemical intermediates

*Linde Schoenmaker[1,2], Daan A. Jiskoot[1,2†], Jenke Scheen[3], Evien Cheng[2], Vytautas Gapsys[4], David F. Hahn[4], Benjamin Ries[5,6], Gerard J.P. van Westen[1], David L. Mobley[2], and Willem Jespers[1]**

1. Leiden Academic Centre of Drug Research, Leiden University, Einsteinweg 55, 2333 CC Leiden, The Netherlands
2. Department of Pharmaceutical Sciences, University of California, Irvine, California 92697, United States
3. Open Molecular Software Foundation, Davis, California 95618, United States
4. Computational Chemistry, Janssen Research and Development, Janssen Pharmaceutica N. V., Turnhoutseweg 30, 2340 Beerse, Belgium
5. Boehringer Ingelheim Pharma GmbH & Co KG, Medicinal Chemistry, Birkendorfer Str 65, 88397 Biberach an der Riss, Germany
6. Open Free Energy, Open Molecular Software Foundation, Davis, California 95616, United States

SI Table 1. Mean and standard deviation values of ΔG of 3 replicates run in protein, water and vacuum.

	Protein (mean)	Protein (std)	Water (mean)	Water (std)	Vacuum (mean)	Vacuum (std)
1PA->1PB	37.47	0.71	44.57	0.11	46.19	0.1
1PA->1I0	4.87	0.71	5.44	0.06	6.33	0.05
1PB->1I0	4.43	0.09	2.9	0.06	1.94	0.08
1PA->1I0->1PB	0.43	0.71	2.54	0.09	4.39	0.1
1PA->1I1	8.46	0.53	9.54	0.06	10.72	0.04
1PB->1I1	-0.84	0.56	-3.83	0.05	-4.49	0.14
1PA->1I1->1PB	9.3	0.77	13.37	0.08	15.21	0.15
2PA->2PB	15.47	0.55	8.95	0.17	11.53	0.34
2PA->2I0	18.93	0.39	14.85	0.05	14.42	0.12
2PB->2I0	8.27	0.22	8.46	0.05	5.99	0.02
2PA->2I0->2PB	10.66	0.45	6.39	0.07	8.43	0.12
2PA->2I1	-2.61	0.19	-4.02	0.17	-0.83	0.2
2PB->2I1	8.37	0.44	9.57	0.04	10.38	0.12
2PA->2I1->2PB	-10.98	0.48	-13.59	0.18	-11.21	0.24
3PA->3PB	35.27	0.34	30.1	0.19	34.08	0.62
3PA->3I0	18.26	0.98	14.88	0.08	14.65	0.04
3PB->3I0	11.59	0.3	12.06	0.04	8.6	0
3PA->3I0->3PB	6.68	1.02	2.81	0.09	6.05	0.04
3PA->3I1	15.38	0.6	16.26	0.07	19.58	0.1
3PB->3I1	11.47	0.44	6.48	0.2	6.82	0.17
3PA->3I1->3PB	3.91	0.74	9.79	0.21	12.75	0.2
4PA->4PB	4.11	0.94	-4.71	0.12	-2.59	0.21
4PA->4I0	13.89	0.82	13.18	0.21	15.18	0.09
4PB->4I0	3.64	0.61	13.65	0.45	13.92	0.08
4PA->4I0->4PB	10.25	1.02	-0.47	0.5	1.26	0.12

4PA->4I1	3.86	0.5	-5.86	0.34	-3.26	0.12
4PB->4I1	1.24	0.53	0.11	0.13		
4PA->4II->4PB	2.62	0.73	-5.98	0.37	-3.26	0.12
5PA->5PB	-4.75	0.41	-4.94	0.05	-6.25	0.02
5PA->5I0	6.78	0.4	4.51	0.04	1.23	0.02
5PB->5I0	-21.07	0.91	-20.33	0.01	-22.13	0.02
5PA->5I0->5PB	27.85	0.99	24.84	0.04	23.36	0.03
5PA->5I1	-9.8	0.18	-9.47	0.02	-7.59	0.03
5PB->5I1	-2.85	0.13	-1.12	0.03	2.13	0.03
5PA->5I1->5PB	-6.95	0.22	-8.35	0.03	-9.72	0.04
6PA->6PB	13.85	1.11	11.27	0.06	12.97	0.21
6PA->6I0	-6.35	0.37	-9.68	0.07	-9.88	0.08
6PB->6I0	16.19	0.61	20.38	0.18	18.33	0.2
6PA->6I0->6PB	-22.54	0.71	-30.06	0.2	-28.2	0.21
6PA->6I1	14.55	0.49	14.77	0.2	17.69	0.14
6PB->6I1	22.71	0.12	16.36	0.11	17.57	0.19
6PA->6I1->6PB	-8.16	0.5	-1.59	0.23	0.13	0.24
7PA->7PB	3.39	1.21	2.76	0.05	0.38	0.31
7PA->7I0	3.23	0.43	2.46	0.04	2.3	0.06
7PB->7I0	4.28	0.07	1.46	0.07	2.99	0.06
7PA->7I0->7PB	-1.04	0.44	1	0.08	-0.69	0.09

SI Table 2. Mean and standard deviation values of RHFE and RBFE ΔΔG of 3 replicates.

	ΔΔG RHFE (mean)	ΔΔG RHFE (std)	ΔΔG RBFE (mean)	ΔΔG RBFE (std)
1PA->1PB	-1.62	0.15	-7.10	0.72
1PA->1I0	-0.89	0.08	-0.57	0.71
1PB->1I0	0.96	0.10	1.53	0.11
1PA->1I0->1PB	-1.85	0.13	-2.11	0.72
1PA->1I1	-1.18	0.08	-1.08	0.53

1PB->1I1	0.66	0.15	2.99	0.56
1PA->1I1->1PB	-1.84	0.17	-4.07	0.78
2PA->2PB	-2.58	0.38	6.52	0.57
2PA->2I0	0.43	0.12	4.08	0.40
2PB->2I0	2.46	0.06	-0.19	0.23
2PA->2I0->2PB	-2.03	0.14	4.27	0.46
2PA->2I1	-3.19	0.27	1.41	0.26
2PB->2I1	-0.80	0.13	-1.20	0.44
2PA->2I1->2PB	-2.38	0.30	2.60	0.51
3PA->3PB	-3.98	0.65	5.17	0.38
3PA->3I0	0.22	0.09	3.38	0.98
3PB->3I0	3.46	0.04	-0.48	0.30
3PA->3I0->3PB	-3.24	0.10	3.86	1.03
3PA->3I1	-3.31	0.12	-0.89	0.60
3PB->3I1	-0.35	0.26	4.99	0.48
3PA->3I1->3PB	-2.97	0.29	-5.88	0.77
4PA->4PB	-2.11	0.25	8.82	0.95
4PA->4I0	-2.00	0.23	0.70	0.84
4PB->4I0	-0.28	0.46	-10.01	0.76
4PA->4I0->4PB	-1.72	0.52	10.71	1.14
4PA->4I1	-2.61	0.36	9.73	0.60
4PB->4I1	0.11	0.13	1.13	0.55
4PA->4II->4PB	-2.72	0.38	8.60	0.81
5PA->5PB	1.31	0.05	0.19	0.41
5PA->5I0	3.28	0.05	2.27	0.40
5PB->5I0	1.80	0.02	-0.74	0.91
5PA->5I0->5PB	1.48	0.05	3.01	0.99

5PA->5I1	-1.88	0.04	-0.33	0.18
5PB->5I1	-3.25	0.04	-1.73	0.14
5PA->5I1->5PB	1.37	0.06	1.40	0.23
6PA->6PB	-1.70	0.22	2.58	1.11
6PA->6I0	0.20	0.11	3.33	0.38
6PB->6I0	2.05	0.27	-4.19	0.63
6PA->6I0->6PB	-1.85	0.29	7.52	0.74
6PA->6I1	-2.92	0.24	-0.22	0.53
6PB->6I1	-1.21	0.22	6.35	0.16
6PA->6I1->6PB	-1.71	0.33	-6.57	0.55
7PA->7PB	2.39	0.32	0.63	1.21
7PA->7I0	0.16	0.07	0.77	0.44
7PB->7I0	-1.53	0.10	2.82	0.10
7PA->7I0->7PB	1.69	0.12	-2.05	0.45
